# Cytotoxic Effect of Ethanol Extract of Microalga, *Chaetoceros calcitrans*, and Its Mechanisms in Inducing Apoptosis in Human Breast Cancer Cell Line

**DOI:** 10.1155/2013/783690

**Published:** 2012-12-30

**Authors:** Siyamak Ebrahimi Nigjeh, Fatimah Md Yusoff, Noorjahan Banu Mohamed Alitheen, Mehdi Rasoli, Yeap Swee Keong, Abdul Rahman bin Omar

**Affiliations:** ^1^Institute of Bioscience, Universiti Putra Malaysia, 43400 UPM Serdang, Selangor, Malaysia; ^2^Department of Aquaculture, Faculty of Agriculture, Universiti Putra Malaysia, 43400 UPM Serdang, Selangor, Malaysia; ^3^Faculty of Biotechnology and Biomolecular Sciences, Universiti Putra Malaysia, 43400 UPM Serdang, Selangor, Malaysia; ^4^Faculty of Veterinary Medicine, Universiti Putra Malaysia, 43400 UPM Serdang, Selangor, Malaysia

## Abstract

Marine microalgae have been prominently featured in cancer research. Here, we examined cytotoxic effect and apoptosis mechanism of crude ethanol extracts of an indigenous microalga, *Chaetoceros calcitrans *(UPMAAHU10) on human breast cell lines. MCF-7 was more sensitive than MCF-10A with IC50 value of 3.00 ± 0.65, whilst the IC50 value of Tamoxifen against MCF-7 was 12.00 ± 0.52 **μ**g/mL after 24 hour incubation. Based on Annexin V/Propidium iodide and cell cycle flow cytometry analysis, it was found that inhibition of cell growth by EEC on MCF-7 cells was through the induction of apoptosis without cell cycle arrest. The apoptotic cells at subG0/G1 phase in treated MCF-7 cells at 48 and 72 hours showed 34 and 16 folds increased compared to extract treated MCF-10A cells which showed only 6 and 7 folds increased at the same time points, respectively. Based on GeXP study, EEC induced apoptosis on MCF-7 cells via modulation of CDK2, MDM2, p21Cip1, Cyclin A2, Bax and Bcl-2. The EEC treated MCF-7 cells also showed an increase in Bax/Bcl-2 ratio that in turn activated the caspase-dependent pathways by activating caspase 7. Thus, marine microalga, *Chaetoceros calcitrans *may be considered a good candidate to be developed as a new anti-breast cancer drug.

## 1. Introduction

Breast cancer is the most prevalent cancer in the world (22% of all the cases) and causes the highest percentage of the cancer deaths (14% of all cancer deaths) in women worldwide. In fact, it is the most common female cancer in both developing and developed countries [1]. There are different options of treatment for breast cancer like surgery, radiation therapy, hormone therapy, chemotherapy, and targeted therapy but these therapies are also associated with some serious side effects. Algae have been widely used by millions of humans and animals around the world as nutritional or pharmaceutical ingredient. Many studies have shown that some algae contain various biologically active substances with potential therapeutic applications in humans [2]. Due to the diverse structural forms and biological activities of marine microalgae, they can be used as a valuable source of molecules for new drug development, including novel anticancer compounds [3]. Indigenous microalgae from Malaysia such as *Chaetoceros calcitrans* have been shown to be potential sources of high-value chemicals including polyunsaturated fatty acids, carotenoids, phycocyanin, and phycoerythrin. All of these active ingredients indicate that such microalgae are a potential source of natural antioxidant and may show anti-inflammatory and cytotoxic effects [4]. This study was aimed to illustrate the cytotoxic and apoptosis effects of ethanol crude extract of an indigenous microalga, *Chaetoceros calcitrans*, (EEC) in human breast cell line, MCF-7. We have further measured the changes of apoptotic related genes such as Bax, Bcl-2, BCL2L1, TNF, P53, Fas, Casp3, Casp7, and Casp9 and cell cycle related genes such as P21, Cyclin A2, CDK2, and MDM2 using GeXP assay. Based on the result in this study, microalgae possessed the potential as sources of anticancer agent.

## 2. Materials and Methods

### 2.1. **Microalgae Strain and Culturing of *Chaetoceros calcitrans***

A pure culture of an indigenous microalga *Chaetoceros calcitrans* (UPMAAHU10) was cultured in 250 to 500 mL of Conway media incubated at ambient temperature of 24 ± 2°C under constant light of 120 *μ*mol photons m² sec^−1^ in an automatic oscillating shaker at 110 rpm. Meanwhile, cultures of 5 litters were maintained in flasks at similar condition with aeration.

### 2.2. Preparation of Microalgae Extract

Microalga culture reaching stationary phase (6-7 days) was harvested by centrifugation at 3500 rpm for 8 min. Resulting pellets were washed with 0.5 M ammonium formate (Sigma-Aldrich, USA) to remove excess salt. Concentrated microalgae cells were collected and dried in 40°C incubator for 3 days. The cells were homogenized with 100% ethanol (Sigma-Aldrich, USA) for 4 days. After that, the supernatant was filtered using filter cotton and 0.2 *μ*m filtration unit (Millipore, Bedford, Japan). Supernatant was then rotary evaporated under reduced pressure at 30°C (Buchi Rotavapor R 200; Buchi Labortechnik, Flawil, Switzerland) to remove all the ethanol, as described by Lee et al. [5]. The extracts were weighted and mixed with DMSO and kept in −20°c until further use.

### 2.3. Cell Cultures

The MCF-7 cells were cultured in DMEM medium with L-glutamine (Gibco, USA), supplemented with 10% (v/v) fetal bovine serum (FBS) and Penicillin (100 U/mL)/Streptomycin (100 U/mL), (Gibco, USA). The MCF-10A cells were maintained with DMEM-F12 medium, (Gibco, USA), supplemented with 10% FBS, HEPS, Glutamine, and Penicillin (100 U/mL)/Streptomycin (100 U/mL), (Gibco, USA). For the preparation of peripheral blood mononuclear cells (PBMC), 10 mL of whole blood from healthy individual were taken by using a 10 mL single-use syringe (Omnifix, Germany), mixed, and diluted with the same volume of phosphate buffer saline (PBS). Samples were centrifuged on Ficoll-Paque Plus (Amersham Biosciences, USA) at 400 ×g for 40 minutes. Interface containing lymphocytes were collected, washed, and pelleted down with PBS. Finally, the supernatant was removed and the pellet was mixed with 2 mL RPMI-1640 media with 10% fetal bovine serum (FBS). The trypan blue dye (Sigma-Aldrich, USA) exclusion assay was performed to determine cell number and viability.

### 2.4. Cytotoxic Assay (MTT Assay)

Cytotoxicity effect of EEC was determined by MTT assay, as previously described by Mosmann [6]. Briefly, MTT solution (Sigma-Aldrich, USA) was dissolved in PBS at 5 mg/mL. Twenty *μ*L of 5 *μ*g/mL MTT solutions was added directly to all appropriate wells. Cells were plated in 96-well plates at an initial density of 1 × 10^5^ cells/mL. After incubation for 24 h at 37°C, cells were treated with various concentrations of EEC and incubated for 24, and 72 hours. MTT solution was added to each well and further incubated for 4 h at 37°C. The optical density was read with an ELISA reader (Bio-Tek Instruments, USA) at 570 nm. Each concentration of the algal extract was assayed in triplicate. IC50 values were determined by plotting a linear regression curve. The percent cell viability was calculated as follows:

(1) $$\begin{array}{c} \text{cell}\;\text{ viability}\;\;\left(\%\right)=\frac{\text{OD of treatment}}{\text{OD of control}}\times 100. \end{array}$$

### 2.5. Morphological Study

MCF-7 and MCF-10A cells were treated with IC50 concentration (3.00 *μ*g/mL) of EEC and observed under light microscope after 24 and 72 hours of exposure to the EEC.

### 2.6. Apoptosis Study-Annexin V/Propidium Iodide

The Annexin V/Propidium Iodide assay was performed according to the manufacturer's recommendation (BD Pharmingen FITC Annexin V Apoptosis Detection Kit). Briefly, MCF-7 cells were plated into a 6-well plates (Nunc, Denmark) and incubated for 12 and 24 hours with ECC at IC50 (3.00 *μ*g/mL) and higher IC50 (3.50 *μ*g/mL) concentrations. The cells at a density of 2.5 × 10^5^ cells/mL were transferred to 1.5 mL centrifuge tube and spun down at 400 × g for 5 minutes at room temperature. The supernatant was discarded and the pellet was incubated in 25 *μ*L of Solution A (trypsin buffer) for 10 minutes at room temperature. Then, 200 *μ*L of Solution B (trypsin inhibitor and RNase buffer) was added to each tube and incubated for 10 minutes at room temperature. After that 200 *μ*L of cold Solution C (propidium iodide stain solution) was added to each tube and gently mixed by tapping the tube by hand, incubated for 10 minutes in the dark at 4°C. After filtration the samples through 50 *μ*L nylon mesh were analyzed by flow cytometry (BD FACSCalibur, USA). Each sample was tested in triplicate and untreated MCF-7 cells were used as controls.

### 2.7. Cell Cycle Analysis

MCF-7 and MCF-10A cells were incubated with EEC at IC50 concentration for 24, 48, and 72 hours. The cells were then harvested by trypsinization, washed with PBS, and subjected to flow cytometry analysis according to the manufacturer's recommendation (BD CycleTEST PLUS DNA Reagent Kit). Briefly, cell pellets were fixed with 500 *μ*L iced cold 80% ethanol and kept at −20°C for 2 hours. The cells were washed twice with PBS, dissolved, and stained in 1 mL PBS buffer containing 0.1% triton X-100, 10 mM EDTA, 50 *μ*g/mL RNase, and 2 *μ*g/mL propidium iodide. The cells were then incubated for 30 minutes at 4°C and were analyzed with flow cytometer (BD FACSCalibur, USA) within 24 hours. Each sample was tested in triplicates and untreated MCF-7 and MCF-10A cells were used as controls.

### 2.8. RNA Extraction

RNA was extracted from the treated and untreated cell lines by using the RNeasy Mini Kit (Qiagen, USA) according to the manufacturer's instructions.

### 2.9. Primer Designing

Primers for a total of 13 target genes, 1 internal control and 2 house-keeping genes were designed using GenomeLab eXpress Profiler software (Table 1). Fragment sizes ranged from 150 to 350 nt with a 7-nt minimum separation size between each PCR product were considered in this study. Kanamycin gene (KAN^r^) was used as an internal control gene, whilst a house-keeping gene, *β*-actin (ACTB) was used as the normalization gene.

### 2.10. cDNA Synthesis and PCR Amplification

The reverse transcription reactions and PCR amplification were performed according to the GenomeLab GeXP Start Kit using the manufacturer's protocols (Beckman Coulter, USA).

### 2.11. GeXP Multiplex Data Analysis

The GeXP system was used to separate PCR products based on size by capillary gel electrophoresis and to measure their dye signal strength in arbitrary units of optical fluorescence, defined as the fluorescent signal minus background. PCR product sizes were determined using GenomeLab GeXP software and were compared to the expected PCR product size to identify each transcript. The data were imported into the analysis module of eXpress Profiler software. The housekeeping gene ACTB was used to normalize the results from each gene. The gene expression data were normalized by dividing the peak area of each gene by the peak area of the ACTB gene and the fold change of expression of each gene was calculated using the following formula: fold change = normalized data of the gene from treated samples/normalized data of the gene from untreated samples. The data for each gene and technical replicate were averaged and calculated.

### 2.12. Statistical Analysis

All data were expressed as means ± SEM of at least three independent experiments. Data were analyzed by ANOVA and Duncan grouping was performed using the SAS system program version 16.0 to identify significant differences between samples. Differences were considered to be significant when *P* < 0.05. Dose response curves were plotted and the IC50 values (concentrations at which cellular effects are inhibited by 50%) were calculated using a linear regression analysis.

## 3. Results

### 3.1. Cell Viability Study (MTT Assay)

Cytotoxic effect of ethanol extract from *Chaetoceros calcitrans* (EEC) was tested against MCF-7 and MCF-10A cells using colorimetric method MTT assay. Tamoxifen was used as a standard to compare with the EEC tested against MCF-7 cells. Furthermore, the effect of EEC was tested on PBMC to determine the cytotoxicity effect of EEC on human normal peripheral blood mononuclear cells. Bioactivity of EEC was determined based on the concentration that induced 50% inhibition on the growth of the treated cells as compared to the controls in triplicate. MCF-7 and MCF-10A cells were exposed to various concentrations of EEC (0 to 30 *μ*g/mL) for 24 and 72 hours. The IC50 values of EEC on MCF-7 and MCF-10A cells were 3.00 ± 0.65 and 12.00 ± 0.59 *μ*g/mL for 24-hour exposure, respectively. A further reduction in IC50 values of EEC was observed after treatment at 72 hour on MCF-7 and MCF-10A cell lines, 2.69 ± 0.24 and 3.30 ± 0.36 *μ*g/mL, respectively. The IC50 values of Tamoxifen on MCF-7 cells at 24 and 72 hours were 12.00 ± 0.52 and 9.00 ± 0.40 *μ*g/mL, respectively (Table 2). Furthermore, EEC did not show cytotoxic effect on PBMC at different concentrations (Table 2).

### 3.2. Morphological Study

**Figure 1:**
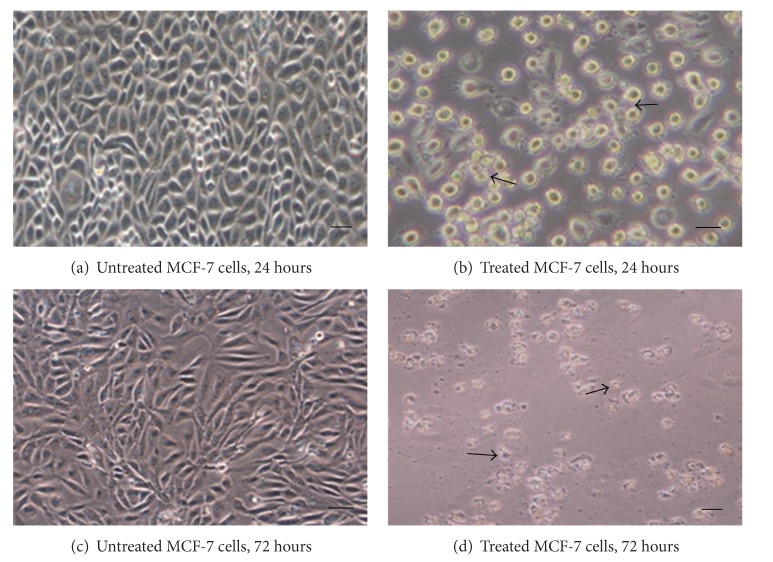
Morphology changes of MCF-7 cells after EEC treatment for 24 (a) and 72 (c) hours. Treated cells were rounded up and cell to cell adhesion was lost. Arrows show membrane blebbing and rounded cells at 40x magnification. The black bar in each image represents 10 *μ*m.

The EEC treated MCF-7 cells became rounded up, shrunken in size, and detached from the monolayer surface of the wells (Figure 1). Number of cells was also found decreased when compared to the control and some EEC treated cells showed membrane blebbing and formation of apoptotic bodies which appeared to be round or oval masses of cytoplasm smaller than the original cell.

### 3.3. Annexin V/Propidium Iodide Study

**Figure 2:**
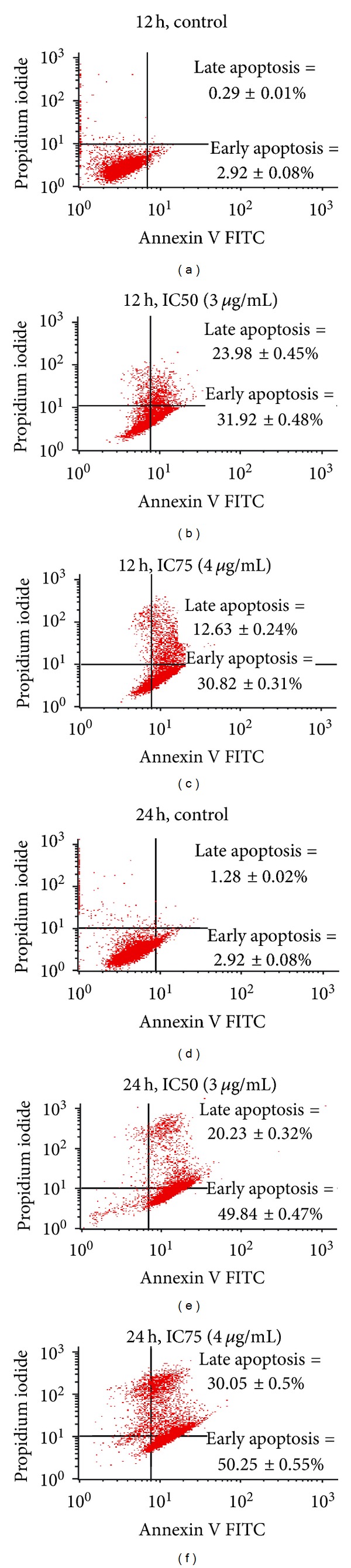
Annexin V/PI study on MCF-7 cell treated with IC50 and IC75 of EEC for 12 and 24 hours.

A significant difference (*P* < 0.001) in viability, early apoptosis, and late apoptosis of MCF-7 cells were detected after treatment with EEC for 12 hours at IC50 concentration (3.00 *μ*g/mL) as compared to the control (Figure 2). Incubation of MCF-7 with IC50 concentration of EEC after 12 hour reduced the cell viability to 26.45 ± 0.41% with 31.92 ± 0.48% and 23.98 ± 0.45% of early apoptosis and late apoptosis, respectively. When the EEC concentration was increased to IC75 (4 *μ*g/mL), a further reduction in cell viability to 23.99 ± 0.33% with 30.82 ± 0.31 and 20.23 ± 0.32% of early apoptosis and late apoptosis in MCF-7 cells, respectively, was detected.

A similar reduction in cell viability and increase in apoptotic cells were detected in MCF-7 cells after EEC incubation for 24 hours. The control untreated cells showed 86.62 ± 0.23%, 8.62 ± 0.19%, and 1.28 ± 0.02% of viability, early apoptosis, and late apoptosis, respectively. However, incubation of MCF-7 with IC50 concentration of EEC after 24 hours reduced the cell viability to 31.61 ± 0.24% with 49.84 ± 0.47% and 12.63 ± 0.24% of early apoptosis and late apoptosis, respectively (Figure 2). In comparison, the higher doses (4 *μ*g/mL) of EEC showed 14.05 ± 0.22%, 50.25 ± 0.55%, and 30.05 ± 0.50% of viability, early apoptosis, and late apoptosis, respectively, in MCF-7 cells after 24-hour incubation. Only the percentages of viable and late apoptotic between the two different concentrations were significantly different, indicating that increasing the concentration of EEC reduced the viable cells, and increased the number of cells that underwent late apoptosis at 24 hour incubation time. Furthermore, when the proportion of normal and apoptotic cells was scored as a percentage of the total cell population, viable cells decreased from 91% before treatment to less than 26% after 12 hours and 32% after 24 hours. Meanwhile the apoptotic cells increased from 0.3% before treatment to 24% and 13% after 12 and 24 hours of incubation, respectively.

### 3.4. Cell Cycle Analysis

In cells treated with EEC, a sub-population of cells appears before the G1 peak is referred to as the subG1 peak. Results indicated that the subG1 population, which indicated apoptotic cells [7], increased from 0.39% in the control (untreated cells) to 2.31% after exposure to 3.00 *μ*g/mL of the EEC for 24 hours. The subG1 population increased further from 1.62% for the control cells to 55.75% after exposure to EEC for 48 hours and from 4.53% for the control cells to 72.75% after exposure to EEC for 72 hour (Table 3). EEC treated MCF-10A cells also showed similar patterns with a less significant effect where the subG0-G1 population increased from 4.10% in the control to 17.52% after exposure to 3.00 *μ*g/mL of EEC for 24 hours. Similarly, the subG0-G1 population increased from 4.50% in the control cells to 27.24% after exposure to EEC for 48 hours and also increased from 5% in the control cells to 37.18% after exposure to EEC for 72 hours (Table 3).

### 3.5. Gene Expression Study

**Figure 3:**
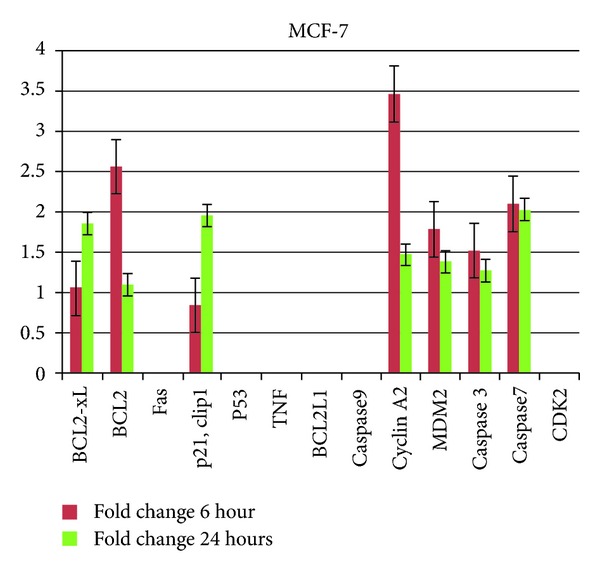
Fold change analysis of gene expressions in MCF-7 cells after 6- and 24-hour treatment with EEC. The lowest fold change 0.8 in p21Cip1 and the highest fold change 3.4 in Cyclin A2 at 6 hours of treatment.

The differential expressions of genes in MCF-7 and MCF-10A cells after treatment were compared with the controls (untreated MCF-7 and MCF-10A cells) and expressed as fold change. As shown in Figure 3, the fold changes of MDM2 and Cyclin A2 in MCF-7 cells decreased from 1.8 and 3.5 at 6 hours to 1.4 and 1.5 at 24 hours, respectively. Meanwhile, the fold change of p21Cip1 increased from 0.8 at 6 hours to 1.9 at 24 hours. An increase in fold change of proapoptotic gene, Bax, from 1.1 at 6 hours to 1.9 at 24 hours was also detected from the treated MCF-7 cells. In contrast, the fold change of antiapoptotic gene, Bcl-2, decreased from 2.5 at 6 hours to 1.1 at 24 hours. The EEC treated MCF-7 cells also showed a decrease in fold changes of effector caspase, where caspase 3 and caspase 7 in treated MCF-7 decreased from 1.5 and 2.1 at 6 hour to 1.3 and 2.0 at 24 hours, respectively.

**Figure 4:**
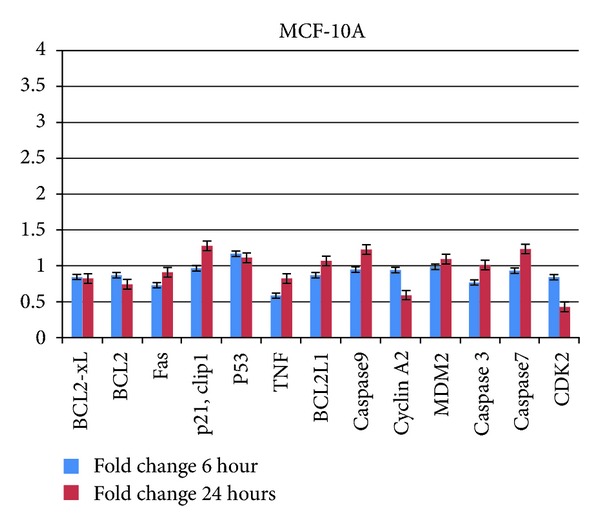
Fold change analysis of gene expressions in MCF-10A cells after 6- and 24-hour treatments with EEC. The lowest fold change 0.4 in CDK2 and the highest fold change 1.3 in p21Cip1 at 24 hours of treatment.

As shown in Figure 4, the fold changes of all the genes in MCF-10A cells were not more than 1.0 except for p53 and MDM2 at both time points 6 and 24 hours as well as for p21Cip1, BCL2l1, caspase 3, caspase 7 and caspase 9 at 24 hours. The fold changes of Bax, Bcl-2, p53, Cyclin A2, and CDK2 decreased after 24-hour incubation time compared to 6 hour samples. Meanwhile, the fold changes for other genes such as p21Cip1, Fas, TNF alpha, MDM2, BCL2L1, caspase 3, 7, and 9 increased at 24-hour incubation time compared to 6 hour samples.

## 4. Discussion

In this study, the ethanol extract from *Chaetoceros calcitrans* (EEC) was extracted and tested on human breast cancer cell lines. In general, EEC showed different IC50 values on the tested cell lines, MCF-7 and MCF-10A at different time points. However, MCF-7 was more responsive to the EEC than MCF-10A with IC50 value of 3.00 ± 0.65. Based on MTT assay, EEC can be considered as potential cytotoxic agent because it showed four fold cytotoxic effect on MCF-7 compared to MCF-10A, with no significant effects on PBMC. This result confirmed the earlier study that reported on the potential antiproliferative effect of five *Dunaliella salina* ethanol extract on AML cell lines [8].

Apoptosis, or programmed cell death, is characterized by a number of well-defined features which include condensation and fragmentation of the chromatin, internucleosomal DNA cleavage, membrane blebbing, caspase activation, and translocation of phosphatidylserine from the inner to the outer leaflet of the plasma membrane [9]. Hence, induction of apoptosis is one of the useful approaches in cancer therapies [10]. Based on Annexin V/PI and cell cycle flow cytometry analysis, we found that inhibition of cell growth by EEC on MCF-7 cells is through the induction of apoptosis without cell cycle arrest. In a previous study, the ethanolic extracts of *Corallina pilulifera* was reported to induce apoptosis in HeLa cells without cell cycle arrest [11, 12]. Even though the EEC treated MCF-10A cells undergo apoptosis, the percentages of apoptotic cells are lower compared to MCF-7 cells. The apoptotic cells at subG0/G1 phase in treated MCF-7 cells at 48 and 72 hours showed 34- and 16-fold increase compared to EEC treated MCF-10A cells which showed only 6- and 7-fold increase at the same time points, respectively. Other study demonstrated that the methanolic extracts of *Plocamium telfairiae* induce apoptosis in HT-29 human colon carcinoma cells [13].

MCF-10A is a nontumorigenic mammary epithelial cell line [14]. The expression profiles of apoptotic genes in human breast cancer lines including the normal human breast cell line, MCF-10A cells, have been studied by several investigators [15, 16]. In this study, we analyzed the expression of 13 apoptotic and cell cycle related genes following treatment with EEC on MCF-7 and MCF-10A cells. The expression levels of all the genes in MCF-10A were detected and the majority of them did not show significant variation in gene expression where the lowest and the highest fold changes were 0.4 and 1.3 for CDK2 and p21Cip1, respectively. However, the fold change of MDM2 in EEC treated MCF-7 cells was 1.8 and 1.4 at 6 and 24 hours, respectively (Figure 3). Hence, the result supported an earlier study indicating that there is a direct link between MDM2 expression and programmed cell death [17]. Moreover, the fold change of Cyclin A2 expression in MCF-7 cells decreased from 3.5 after 6 hours of exposure to 1.5 after 24 hours. Besides that, the fold changes of p21Cip1 in treated MCF-7 cells were 0.8 and 1.9 after 6 and 24 hours of exposure to EEC, respectively (Figure 3). The function of CDKs is tightly regulated by cell cycle inhibitors like p21Cip1 and p27Kip1 [18] where uncontrolled CDK activity is usually the reason of cancer. p21Cip1 protein binding inhibits the activity of cyclin-CDK2 complexes. This protein was reported to be specifically cleaved by caspase 3 like caspases which cause the activation of CDK2 and may be instrumental in the execution of apoptosis following activation. Other studies have shown that the growth inhibitory effect of astaxanthin-rich *H*. *pluvialis* extract on HCT-116 colon cancer cells was associated with an increase in p21Cip1 expression, cell cycle arrest, and the induction of apoptosis [17].

Besides that, the fold change of proapoptotic gene, Bax, expression in treated MCF-7 cells for 6 and 24 hour exposed samples was 1.1 and 1.9, respectively (Figure 3). Bax gene is a member of the Bcl-2 family, an apoptosis promoter that regulates the release of cytochrome *c* from mitochondria, and its expression is identified to lead to the activation of caspases and programmed cell death [19]. Besides that, an earlier study has shown that the ethanol extract of *Dunaliella salina* induced apoptosis of A549 human lung cancer cell line by elevating Bax expression [20]. Meanwhile, acetone extract of *Lethariella zahlbruckneri* increased the expression of the proapoptotic protein, Bax, and decreased the expression of the antiapoptotic protein, Bcl-2 [21]. As shown in Figure 3, the fold changes of Bcl-2 in MCF-7 cells were 2.5 and 1.1 at 6 and 24 hours, respectively. More importantly, increasing the incubation time from 6 to 24 hours led to the increase of Bax/Bcl-2 ratio, which is an important apoptosis inducer indicator in cancer cells. Our results supported a previous study that demonstrated the inducing of apoptosis of astaxanthin-rich *Haematococcus pluvialis* extract on HCT-116 colon cancer cells by the increasing of the ratio of Bax/Bcl-2 expression [22]. Overexpressions of Bcl-2 and Bcl-xl have been demonstrated in a large variety of human malignancies, including breast, prostate, colorectal, and lung cancers [23]. The ability of tumour cells to undergo apoptosis by chemotherapeutic agents is controlled by the ratio of Bax/Bcl-2 in the mitochondria [24]. The Bax activation might have involved in the release of cytochrome c from the mitochondria and clustered with APAF-1, an apoptotic protease activating factor 1 and resulted in activation of caspase 9 which then cleaved the downstream caspease 3, 6, and 7 that led to apoptosis [25]. Figure 3 showed upregulation of effectors caspase, caspase 3, and caspase 7 in treated MCF-7 at 6 hour (1.5 and 2.1) and 24 hours (1.3 and 2.0), respectively. Results from this study concur with a previous study that demonstrated chloroform extract of *Physalis minima* produced a significant growth inhibition and induced apoptosis against human MCF-7 by activation of caspase 3 [14]. Thus, activation of caspase is recognized to be the most specific indication of apoptosis [23].

Based on the results obtained from this study, it postulates that EEC could induce apoptosis through a caspase-dependent pathway by activating caspase 3 and 7 in MCF-7 cells. Hence, Bax activation might have involved in the release of cytochrome c from the mitochondria and probably clustered with APAF-1 and resulted in activation of caspase 9 which then cleaved the downstream caspase 3, 6, and 7 that led to apoptosis. Thus, this study concluded that the crude ethanol extracts of *C*. *calcitrans* has the potential to be used as therapeutic and chemopreventive agents for breast cancer treatment.

## Figures and Tables

**Table 1:** Selected apoptotic and cell cycle related genes used in GeXP assay.

Gene	Accession number	Product size	Left sequence*	Right Sequence**
BCL2	M14745	157	ACCACTAATTGCCAAGCACC	TTTTCCATCCGTCTGCTCTT
Fas	NM_000043	165	CTCCAAGGGATTGGAATTGA	TGCAGTCCCTAGCTTTCCTT
TNF alpha	NM_000594	171	CTATCTGGGAGGGGTCTTCC	ATGTTCGTCCTCCTCACAGG
Caspase 3	NM_004346	182	GAACTGGACTGTGGCATTGA	ACCAGGAGCCATCCTTTGA
p21Cip1	NM_000389	202	TGTGGACCTGTCACTGTCTTG	TAGGGCTTCCTCTTGGAGAA
Cyclin A2	NM_001237	212	TATTGCTGGAGCTGCCTTTC	CTTTTCTCTTATTGACTGTTGTGCAT
BCL2L1	NM_001191	232	CCACAGCAGCAGTTTGGAT	GGGATTGTTCCCATAGAGTTCCACAA
MDM2	NM_002392	239	GGTGGGAGTGATCAAAAGGA	ACCAGGCTTTCATCAAAGGAA
Caspase 7	NM_033340	247	CAGACCGGTCCTCGTTTGTA	ACCTCGGCATCTTTGTCTGTT
CDK2	NM_052827	285	TGGTGGCGCTTAAGAAAATC	ACAGCTGGAACAGATAGCTCTTGA
ACTB^a^	NM_001101	295	CTGGCACCACACCTTCTACA	AAGGGCATACCCCTCGTAGAT
Bax	BC014175	316	CCCTTTTGCTTCAGGGTTTC	ACAAAGTAGAAAAGGGCGACAA
KAN^b^	Kan(r)	325	ATCATCAGCATTGCATTCGATTCCTGTTTG	AATTCCGACTCGTCCAACATC
Caspase 9	NM_001229	332	GGGCTCACTCTGAAGACCTG	ATCTGGAAGCTGCTAAGAGCC
P53	NM_000546	340	TTTTGGGTTTTGGGTCTTTG	ATTCAACATGAGGGACAGCTT

*Forward universal primer sequence (AGGTGACACTATAGAATA).

**Reverse universal primer sequence (GTACGACTCACTATAGGGA).

^a^Gene used for normalization.

^b^Internal control.

**Table 2:** IC50 of EEC and Tamoxifen on MCF-7 cells.

	IC50 (*μ*g/mL)	
	24 hour	72 hour
EEC treated MCF-7	3.00 ± 0.65	2.69 ± 0.24
EEC treated MCF10A	12.00 ± 0.59	3.30 ± 0.36
EEC treated PBMC	>30	>30
Tamoxifen treated MCF-7	12.00 ± 0.52	9.00 ± 0.40

Values are expressed as mean ± S.E. from triplicate.

**Table:** (a)

	MCF-7 cells Cell cycle phases (Percentage)			
	G0/G1	G2/M	S	SubG0/G1
Control*				
** **24 hours	61.42 ± 0.07	17.12 ± 0.24	22.48 ± 0.19	0.39 ± 0.008
** **48 hours	63.38 ± 0.43	14.19 ± 0.21	14.67 ± 0.40	1.62 ± 0.11
** **72 hours	87.17 ± 0.13	1.63 ± 0.04	6.07 ± 0.03	4.53 ± 0.12
IC50-3 *μ*g/mL				
24 hours	64.82 ± 0.32	12.93 ± 0.56	21.24 ± 0.30	2.31 ± 0.13
48 hours	44.6 ± 1.37^a^	0.01 ± 0.005^a^	0.46 ± 0.02^a^	55.75 ± 1.25^a^
72 hours	27.85 ± 1.26^a^	0.02 ± 0.01	0.35 ± 0.02^a^	72.75 ± 1.23^a^

Values are expressed as mean ± S.E. (Standard Error), *μ*g/mL. (*P* < 0.001, ANOVA).

*Untreated cells were used as a control. ^a^Significant difference with untreated group (*P* < 0.05).

**Table:** (b)

	MCF-10A Cell cycle phases (Percentage)			
	G0/G1	G2/M	S	Sub G0/G1
Control*				
24 hours	63.45 ± 1.70	14.16 ± 1.01	17.20 ± 0.75	4.10 ± 0.14
48 hours	62.25 ± 1.39	12.68 ± 0.51	16.10 ± 0.20	4.50 ± 0.08
72 hours	58.12 ± 1.01	11.38 ± 0.29	20.45 ± 1.22	5.00 ± 0.18
IC50-3 *μ*g/mL				
24 hours	60.49 ± 1.06	3.84 ± 0.12	16.37 ± 0.22	17.52 ± 0.57
48 hours	53.39 ± 0.69	6.4 ± 0.008	12.25 ± 0.29	27.24 ± 0.40
72 hours	44.76 ± 0.77	5.91 ± 0.21	10.63 ± 0.34	37.18 ± 0.53

Values are expressed as mean ± S.E. (Standard Error), *μ*g/mL. (*P* < 0.001, ANOVA).
