# Corrigendum to “Cytotoxic Effect of Ethanol Extract of Microalga, *Chaetoceros calcitrans*, and Its Mechanisms in Inducing Apoptosis in Human Breast Cancer Cell Line”

**DOI:** 10.1155/2016/8693826

**Published:** 2016-11-29

**Authors:** Siyamak Ebrahimi Nigjeh, Fatimah Md Yusoff, Noorjahan Banu Mohamed Alitheen, Mehdi R. Pirozyan, Yeap Swee Keong, Abdul Rahman bin Omar

**Affiliations:** ^1^Institute of Bioscience, Universiti Putra Malaysia, 43400 UPM Serdang, Selangor, Malaysia; ^2^Department of Aquaculture, Faculty of Agriculture, Universiti Putra Malaysia, 43400 UPM Serdang, Selangor, Malaysia; ^3^Faculty of Biotechnology and Biomolecular Sciences, Universiti Putra Malaysia, 43400 UPM Serdang, Selangor, Malaysia; ^4^Faculty of Veterinary Medicine, Universiti Putra Malaysia, 43400 UPM Serdang, Selangor, Malaysia

In the article titled “Cytotoxic Effect of Ethanol Extract of Microalga,* Chaetoceros calcitrans*, and Its Mechanisms in Inducing Apoptosis in Human Breast Cancer Cell Line” [1], the name of the fourth author was given incorrectly as Mehdi Rasoli. The author's name should have been written as Mehdi R. Pirozyan. The revised authors' list is shown above.
